# An Escherichia coli-Induced Distal Arch Aneurysm Presenting With Hemoptysis

**DOI:** 10.7759/cureus.40654

**Published:** 2023-06-19

**Authors:** Hideki Sasaki, Yukihide Numata, Shinji Kamiya, Yoshiaki Sone, Osamu Sasaki

**Affiliations:** 1 Cardiovascular Surgery, Nagoya City University East Medical Center, Nagoya, JPN; 2 Cardiology, Saitama Medical Center, Saitama Medical University, Kawagoe, JPN; 3 Internal Medicine, Kouiki Mombetsu Hospital, Mombetsu, JPN

**Keywords:** emergency department, antibiotics, computed tomography, hemoptysis, infected thoracic aneurysm

## Abstract

Infected thoracic aneurysms are a rare and potentially life-threatening condition that present with non-specific symptoms. We describe here a case of an 83-year-old female who presented to the emergency department with the chief complaint of vomiting blood. The patient had presented to the emergency department 40 days earlier with abdominal pain, fever, and leukocytosis. She had a medical history of traumatic liver injury resulting in bile duct stenosis, necessitating percutaneous transhepatic gallbladder drainage and subsequent bile duct-jejunal anastomosis 25 years ago. Emergency contrast-enhanced computed tomography (CT) revealed an irregular distal arch aneurysm. According to the patient’s present symptoms, CT findings, and medical history, infected thoracic aneurysm was suspected. Total arch replacement was performed promptly, followed by thorough antibiotic therapy. Following successful treatment, the patient’s condition stabilized, and she was transferred to a rehabilitation facility for further recovery.

## Introduction

An infected thoracic aneurysm is a rare and potentially life-threatening condition that presents with a diverse array of symptoms, including fever, chest pain, back pain, and malaise. Due to the rarity of this condition, early recognition and prompt treatment are essential [1]. Accurate diagnosis relies on a thorough evaluation of the patient's medical history, laboratory test results, and imaging studies [2,3]. In this case report, we present the clinical course of a patient who presented to the emergency department with haemoptysis and was ultimately diagnosed with an infected thoracic aneurysm. Through a detailed examination of the patient's history and radiographic studies, we aim to highlight the importance of prompt recognition and treatment of this rare condition.

## Case presentation

This case pertains to an elderly female, aged 83, who was admitted to the emergency department with the chief complaint of vomiting blood. The patient complained of vomiting a small amount of blood for the past two months. Although the amount of vomited blood was not clear, the patient had experienced this episode several times over the past two months. Her medical history was complex, including a diagnosis of diabetes, appendicitis, and a traumatic liver injury resulting in bile duct stenosis, necessitating percutaneous transhepatic gallbladder drainage (PTGBD) and subsequent bile duct-jejunal anastomosis 25 years ago. The patient had presented to the emergency department 40 days back with symptoms of abdominal pain and a fever of 38.2°C, revealing leukocytosis with a count of 14,300/μL and a C-reactive protein level of 5.0mg/dL. Despite the absence of gallbladder stones or bile duct dilation, the attending physician consulted with a gastroenterologist for further evaluation. To address the patient's condition, the gastroenterologist suspected retrograde cholangitis despite the absence of gallbladder stones and bile duct dilatation. A regimen of sulbactam sodium/ampicillin sodium (SBT/ABPC) at a dose of 3g/day was initiated and continued for 10 days. Over time, the patient's symptoms and fever gradually subsided, and she was eventually discharged after a 16-day hospitalization period. However, the patient presented to the emergency department one month later with hematemesis. The laboratory test revealed leukocytosis with a count of 15,000/μL and a C-reactive protein level of 17.32g/dL.

**Figure 1:**
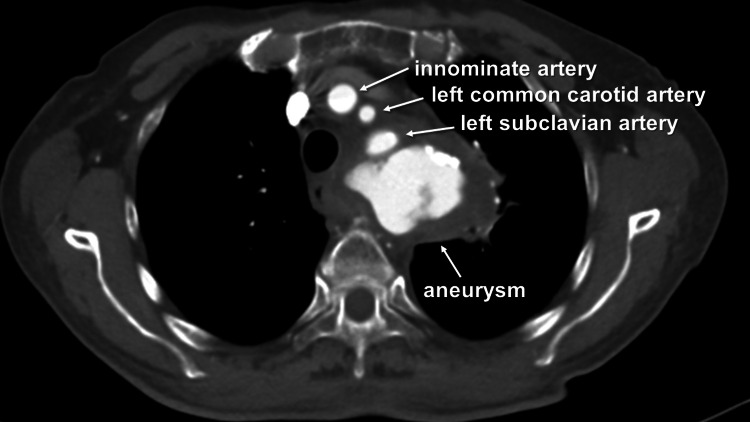
Preoperative contrast-enhanced computed tomography revealing a distal arch aneurysm

A plain computed tomography (CT) scan was ordered, which showed an emergence of an aneurysm at the distal aortic arch compressing the esophagus and the adjoining lung, resulting in atelectasis. Contrast-enhanced CT (CECT) revealed irregular margins of the distal arch aneurysm at various points, prompting consultation with the cardiovascular department (Figure 1).

Furthermore, due to the irregular margin of the adjoining esophagus at some points, the gastroenterology department was consulted. During the upper gastrointestinal endoscopy, conducted by a gastroenterologist, no esophageal bleeding was detected. Nevertheless, the examination identified esophageal compression, which could be attributed to the presence of an aneurysm. Given the inherent risks associated with bronchoscopy, it was not performed, and we speculated that the patient's primary complaint was hemoptysis, likely caused by a ruptured arch aneurysm. Furthermore, considering her medical history and the morphology of the aneurysm, we postulated that it was an infected arch aneurysm, which was later confirmed through blood culture testing revealing the presence of *Escherichia coli*. Following extensive consultation among a team of cardiovascular surgeons, anesthesiologists, and gastroenterologists, a decision was made to proceed with total arch replacement surgery to address the infected arch aneurysm.

**Figure 2:**
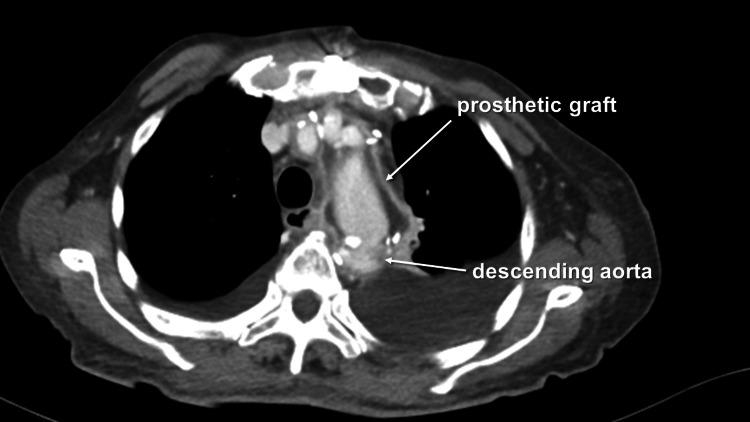
Postoperative enhanced computed tomography revealing no signs of infection recurrence

Under the administration of general anesthesia, a median sternotomy was performed, and a cardiopulmonary bypass (CPB) was established by means of ascending aortic perfusion and right atrium drainage. After the patient's body temperature was reduced to 22°C, circulatory arrest was induced. Cardioplegia was delivered via the aortic root, resulting in cardiac arrest. Subsequently, the aorta was incised towards the aortic arch, and antegrade selective cerebral perfusion was initiated through a balloon-tipped cannula. At the large curvature of the aneurysm, a portion of the aortic wall and adventitia were carefully excised. Fragile tissue was removed, and the distal anastomosis site was trimmed, thoroughly irrigated with saline, and secured with a rifampicin-soaked branched graft, anastomosed to the descending aorta. After the distal anastomosis was completed, the reconstruction of the arch vessels and the proximal anastomosis to the ascending aorta were performed consecutively. The CPB, aortic cross-clamp, and circulatory arrest times were 288 minutes, 225 minutes, and 125 minutes, respectively. The patient was successfully weaned from the CPB and transferred to the intensive care unit (ICU) in a stable condition. The portion of the aneurysmal wall was submitted for culture, which resulted in negative culture findings. After 35 hours post-operation, the patient was successfully weaned off the ventilator and transferred to the ward 48 hours later, following their stay in the ICU. In order to mitigate the risk of bacterial infections recurring, meropenem was administered at a dosage of 2g/day for four days. Subsequently, upon detecting a minimum inhibitory concentration of cefmetazole sodium (CMZ) below 1μg/mL against *Escherichia coli*, a four-week course of CMZ at a dosage of 4g/day was initiated. Throughout this period, the patient's blood tests yielded favorable results, and a postoperative CT scan taken 31 days after the operation scan showed no signs of recurrence of infection in the proximity of the prosthetic graft (Figure 2).

**Figure 3:**
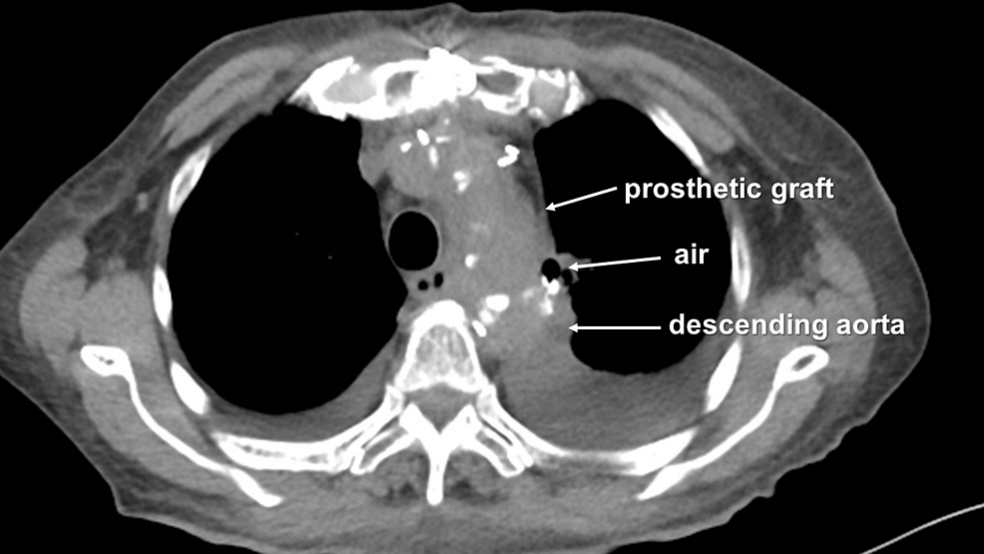
Second postoperative enhanced computed tomography scan revealing air around the prosthetic graft

However, a second postoperative CT scan taken 52 days after the operation detected air surrounding the graft, although the patient's leukocyte count remained stable at 4400/μL and the C-reactive protein level was at 0.92g/dL (Figure 3).

CMZ was discontinued, and tazobactam/piperacillin (TAZ/PIPC) was introduced at a dosage of 13.5g/day for seven days. Notably, a third postoperative CT scan revealed no progression of the condition, and laboratory results indicated that the patient's leukocyte count was 3700/μL and C-reactive protein level was 1.41g/dL, which were deemed within acceptable limits. To sustain their recovery, the patient was prescribed rifampicin (RFP) at a dosage of 450mg/day and minocycline (MINO) at a dosage of 200mg/day, with the intention to continue beyond their transfer to a rehabilitation facility. The patient was eventually transferred to a specialized rehabilitation facility eight days after the third CT scan, with the ultimate objective of achieving further progress and recovery. The patient is currently doing well six months after the operation and is being followed up in the outpatient clinic.

## Discussion

An infected aneurysm is often caused by gram-positive bacteria such as *Staphylococcus* and *Streptococcus *species. It is rare for an infected aneurysm to be caused by *Escherichia coli* [2]. In the present case, *Escherichia coli* infection, proved by the blood culture, contributed to the pathogenesis of the aneurysm. Septicemia with *Escherichia coli* is often related to underlying infections, such as urinary or biliary tract infections [4]. Diagnosing an infected aneurysm can be challenging in clinical settings. Patients with this condition often present with non-specific symptoms, such as fever, dyspnea, general fatigue, and restlessness. While the patient complained of vomiting blood at presentation, this symptom is not specific to an infected aneurysm. Furthermore, after upper gastrointestinal endoscopy revealed no bleeding, it was determined that the patient was actually experiencing hemoptysis, which is also not a specific symptom of an infected aneurysm. In cases where hemoptysis is present, differential diagnoses may include lung cancer, bronchitis, tuberculosis, pneumonia, and chronic obstructive pulmonary disease. Due to the non-specific nature of these symptoms, attending physicians in the emergency department may not initially consider infected aneurysm as a possible diagnosis. It is important for physicians to maintain a high level of suspicion for this condition and to perform a plain CT scan followed by contrast-enhanced CT imaging if necessary. If an aneurysm is located anywhere from the aortic arch to the descending aorta, physicians should consult a cardiovascular surgeon immediately. Hemoptysis is a critical sign of aneurysm invasion into the pulmonary parenchyma, making prompt and accurate diagnosis crucial to the patient's well-being [5]. Although the tissue culture of the aneurysmal wall was negative in the present case, the aneurysm was considered infected due to both a positive blood culture for *Escherichia coli* and the irregular margin of the aneurysm in the CECT. In addition, the patient's past medical history, specifically a traumatic liver injury leading to bile duct stenosis and subsequent procedures such as PTGBD and bile duct-jejunal anastomosis performed 25 years ago, was considered to be associated with *Escherichia coli* infection in the bloodstream, which eventually led to the formation of the infected thoracic aneurysm.

There are two modalities for treating an infected thoracic aneurysm: conventional surgery via median sternotomy or thoracotomy, and endovascular surgery. In the former, as much abscess and infected tissue as possible can be removed, but the surgery using cardiopulmonary bypass may be too invasive for some patients to tolerate. Meanwhile, the latter is a minimally invasive option adopted for elderly, frail, and other patients with comorbidities [2]. However, the infected tissue may remain, and recurrence can be troublesome at times [6].

Hemoptysis is a critical sign of an aneurysm-pulmonary fistula and requires urgent medical attention. While endovascular surgery is a minimally invasive option that can effectively control bleeding in emergency situations, it may not always prevent the condition from recurring. In such cases, radical conventional graft replacement with aneurysm resection may be necessary to achieve a complete cure. Corniquet et al. reported a staged repair for a ruptured infected thoracic aneurysm. They first performed urgent endovascular surgery, followed by allograft repair five days later [3]. A thorough discussion among multidisciplinary professionals is paramount to achieve a favorable outcome.

In the current case, the patient's early clinical course was good, with no problematic signs of infection recurrence in the first postoperative CT scan. However, the second CT scan revealed the presence of air around the artificial graft where the infected aneurysm had been. This CT was scheduled to confirm the absence of infection recurrence around the graft, despite serial laboratory tests indicating gradual improvement of inflammatory indices over time. We had anticipated a negative result, but the presence of air around the graft was unexpected. Nonetheless, blood cultures were negative, and blood chemistry work-up was acceptable. We consulted the infection control team and discontinued CMZ while administering TAZ/PIPC for seven days. Thereafter, the patient received RFP+MINO, which were intended to be continued after discharge. The third CT scan taken 71 days after the operation revealed no signs of infection, although it remained unclear whether the air around the graft had indicated recurrence. While the exact duration for oral antibiotic therapy remains elusive, diligent outpatient monitoring persists, with the patient receiving RFP+MINO.

## Conclusions

Infected thoracic aneurysms are a rare and potentially life-threatening condition that present with non-specific symptoms, emphasizing the need for a high level of suspicion. Hemoptysis accompanied by high-grade fever is one of the symptoms or conditions. When suspected, immediate contrast-enhanced computed tomography is essential for accurate diagnosis. Hemoptysis, a harbinger of impending aneurysmal rupture, warrants prompt surgical intervention followed by a course of antibiotic therapy. Postoperative surveillance with serial computed tomography is essential to detect any recurrence of infection. The appropriate prescription of antibiotics is crucial in order to prevent the recurrence of *Escherichia coli* infection. In conclusion, prompt recognition and management are crucial for ensuring optimal outcomes in patients with infected thoracic aneurysms.
